# High retention and appropriate use of insecticide-treated nets distributed to HIV-affected households in Rakai, Uganda: results from interviews and home visits

**DOI:** 10.1186/1475-2875-8-76

**Published:** 2009-04-22

**Authors:** Lauren Cohee, Lisa A Mills, Joseph Kagaayi, Ilana Jacobs, Ronald Galiwango, James Ludigo, Joseph Ssekasanvu, Steven J Reynolds

**Affiliations:** 1 Johns Hopkins School of Medicine, 1830 East Monument Street #401, Baltimore, MD, USA; 2 Rakai Health Sciences Program, Kalisizo, Uganda; 3 University of Rochester School of Medicine, Rochester, NY, USA; 4 National Institute of Allergy and Infectious Diseases, National Institutes of Health, Bethesda, MD, USA

## Abstract

**Background:**

Distribution of insecticide-treated nets (ITNs) has recently been incorporated into comprehensive care strategies for HIV-positive people in malaria-endemic areas. WHO now recommends free or low-cost distribution of ITNs to *all *persons in malaria-endemic areas, regardless of age, pregnancy and HIV status. Knowledge about and appropriate use of ITNs among HIV-positive ITN recipients and their household members has not been well characterized.

**Methods:**

142 randomly selected adults were interviewed in July–August 2006 to assess knowledge, retention, and appropriate use of ITNs they had received through a PEPFAR-funded comprehensive HIV care programme in rural Uganda.

**Results:**

Among all participants, 102 (72%, CI: 65%–79%) reported they had no ITNs except those provided by the programme. Of 131 participants who stated they were given ≥ 1 ITN, 128 (98%, CI: 96%–100%) stated they still possessed at least one programme-provided ITN. Reported programme-ITN (pITN) use by participants was high: 119 participants (91%, CI: 86%–96%) reported having slept under pITN the night prior to the survey and 115 (88%, CI: 82%–94%) reported sleeping under pITN seven days per week. Being away from home and heat were the most common reasons given for not sleeping under an ITN. A sub-study of thirteen random home visits demonstrated concordance between participants' survey reports and actual use of ITNs in homes.

**Conclusion:**

There was excellent self-reported retention and appropriate use of ITNs distributed as a part of a community-based outpatient HIV care programme. Participants perceived ITNs as useful and were unlikely to have received ITNs from other sources.

## Background

The World Health Organization has recently produced guidelines promoting low-cost or free distribution of long-lasting ITNs for all persons, not just pregnant women, children and HIV-positive persons [1]. In particular, HIV patients in malaria-endemic areas have fewer and less severe episodes of malaria if they use ITNs. [2] It is especially important that pregnant HIV-positive women have access to ITNs. PEPFAR-funded HIV care programmes are now implementing ITN distribution as part of comprehensive HIV care [3]. To ensure maximal household benefit from this intervention, it is essential to understand community perceptions of ITN usefulness and household determinants of who sleeps under ITNs. [4] This study was undertaken in order to assess knowledge, retention and appropriate use of ITNs distributed to HIV-positive adults as a part of a community-based outpatient HIV care programme in Rakai District, Uganda.

HIV prevalence in Rakai District is approximately 12%. The district is has moderately intense seasonal transmission of falciparum malaria, although patterns of rainy and dry seasons have been changing significantly in past years. 41.5% of outpatient visits to clinics and hospitals involve suspected malaria [5]. Baseline ITN use in this region of the country is low: only 13% of households had an ITN in 2004–5 [6]. In Uganda overall, only 11% of pregnant women and children slept under an ITN the night prior to questioning for the most recent national health survey [7]. During the study period, the Uganda Ministry of Health was promoting ITN usage but did not have a distribution programme in Rakai District. Such a program has subsequently begun. Recent studies have suggested progress in ITN household coverage in Uganda, however these may not reflect all regions, especially rural settings such as Rakai District [8].

Approximately 4,000 people are enrolled in the PEPFAR-supported longitudinal HIV outpatient clinic in Rakai District, Uganda. All participants receive cotrimoxazole prophylaxis, general medical care and educational services. Beginning in June 2004, ancillary services included distribution of one ITN. In partnership with Population Services International in December 2004, ancillary services were increased to include two long-lasting ITN (Permanet^® ^impregnated with deltamethrin), such that a total of 3,268 patients had received programme ITNs (pITNs) prior to July 2006. At distribution, participants were educated about how to hang and maintain ITNs by local peer educators or clinic staff. All HIV-positive persons in the household were encouraged to sleep under ITNs at all times of year.

## Methods

From July to August 2006, a survey of 142 adults were randomly selected from the combined roster (including demographic and medical characteristics) of 15 community-based HIV outpatient clinics was performed to assess the effectiveness of a PEPFAR-funded ITN distribution programme. Simple random sampling ensured proportion representation of the sampled clinics which are embedded in communities involved in an ongoing community cohort study. Candidates were omitted from a distant 16^th ^clinic where programme activities were not yet fully operational. Selected participants were approached for interview by clinical research staff in a waiting area prior to clinic visits during dry-season months of typically low malaria transmission in this region. There were no refusals. A standardized questionnaire was used to assess ITN ownership and usage, and household structure.

In addition, approximately 10% of participants (n = 13) who had reported having at least 1 programme ITN were randomly chosen to receive an unannounced home visit including brief interview and examination of household ITNs. A standard data collection form was used which included each participant's responses to the questionnaire previously administered at clinic. Data were entered by study staff using FoxPro, and analysis done in STATA software (Stata Statistical Software: Release 8.0, Stata Corporation, College Station, TX, 2002). 95% Confidence intervals were reported as 'CI' and calculated using the normal approximation interval for all samples except for the home visit results for which Wilson intervals were used because of small sample size.

Participants belonged to the ongoing Rakai Community Cohort Study (RCCS) or the ongoing ARV-related clinical study (ARCS) and all provided consent to have their clinical records used for research. They also provided verbal consent to be interviewed. RCCS and ARCS were both approved by the Johns Hopkins University Western Institutional Review Board, the Science and Ethics Committee of the Uganda Virus Research Institute and the Uganda National Council for Science and Technology.

## Results

### Demographic characteristics and ITN ownership [please see Table 1]

**Table 1:** Ownership and use of ITNs in HIV-affected households

**Participants interviewed**	**N = 142 (100%)**	**95% Conf. Int.**
Female	92/142 (65%)	-

On antiretroviral therapy	112/142 (79%)	-

Household size: <4 individuals	47/142 (33%)	-

4–6 "	71/142 (50%)	-

>6 "	24/142 (17%)	-

Reports having 1 or more ITN in household	138/142 (97%)	(94 – 100)

Mean number of ITNs per person per household	0.8	-

Sleeps under any ITN less than 7 nights/wk (including never)	17~/138 (12%)	(7 – 18)
Why not? Too hot	8/17 (47%)	
ITN unavailable	6/17 (35%)	
Too difficult to mount	1/17 (6%)	
Too few mosquitoes	2/17 (12%)	
Slept away from home	2/17 (12%)	
Forgot	1/17 (6%)	

Sleeps under any ITN 7 nights/wk	124/138 (90%)	(85 – 95)
If occasionally fails to sleep under ITN, why? Too hot	25/124 (20%)	
Slept away from home (work, visits, funerals)	12/124 (10%)	

All ITNs in household were obtained from programme	102/138 (75%)	(65 – 79)

Possessed any ITN from outside source	36/138 (25%)	(19 – 33)

Possessed ITN only from outside sources	7/138 (5%)	(1 – 9)

Source of outside ITNs – purchased	27/36 (75%)	(61 – 89)
Received from non-government agency or relative	9/36 (25%)	(11 – 39)

**Had received 1 or more ITN from programme (pITN)**	131/138 (92%)	(88 – 96)

Still possessed 1 or more pITN	128/131 (98%)	(96 – 100)

Now missing at least 1 pITN previously received:	10/131 (8%)	(3 – 12)
Burned	5/10 (50%)	-
Given away	3/10 (30%)	-
Other	2/10 (20%)	-
Sold	0/10 (0%)	-

At least 1 person slept under pITNs the night prior	123/128 (96%)	(93 – 99)

Did participant sleep under pITNs the night prior?	119*/123 (97%)	(94 – 100)

If participant slept under pITN night prior, uses it 7 nights/week?	115/119 (97%)	(94 – 100)

Who else slept under pITNs the night prior?^®^		
Participant spouses	44	-
Children ≥ 5 years of age	82	-
Children <5 years of age	36	-
Other relatives	14	-
Others	10	-

Households w/≥1 pregnant woman	9/131 (7%)	(3 – 11)

Pregnant woman/women slept under pITNs night prior	5/9 (56%)	(23 – 88)

Believes ITNs are used to prevent malaria	113 (80%)	(73 – 87)

Believes ITNs are useful for keeping out mosquitoes	81 (57%)	(49 – 65)

Believes ITNs are used to prevent fever	26 (18%)	(12 – 24)

~1 participant did not answer

*4 participants did not sleep under pITN but reported that someone in household did

^® ^sum > total because of co-sleeping

Of the 142 participants, 65% were female and 79% were currently taking antiretroviral drugs. 50% lived in households containing 4–6 people, 33% lived in households with less than three people, while 17% lived in household with than six people. One hundred and thirty-eight (97%, CI: 94%–100%) participants stated they had at least one ITN in their household. In total, there was a mean of 0.8 ITNs per person per household. 102 participants (72%, CI: 65%–79%) lived in households where there was no ITN from any source other than our programme. In households with one or more outside ITNs, 75% (CI: 62%–88%) of these had been purchased; the remainder had been given to the participant by either a non-governmental organization or a relative. 131/142 (92%, CI: 88%–96%) participants stated they were given at least one ITN by the programme. 128/131 (98%, CI: 96%–100%) of those that reported being given an ITN stated they still had at least one programme-provided ITN. 73/128 (57%, CI: 48%–66%) of these participants had been given their ITN in the prior six months. Ten participants reported that they no longer had at least one of the programme-provided ITN that had been given to them. Of those ITNs, half were reportedly burned, three were given away, and none was sold.

### Use of programme-provided ITN

123/128 (96%, CI: 93%–99%) of people with programme-provided ITNs reported that at least one person slept under the pITN the night prior to the interview, including 119 participants, 44 participant spouses, 118 participant children, 14 relatives, and 10 unspecified others. Nine households contained one or more pregnant women, and in five of those households, the pregnant woman/women reportedly slept under an ITN. Of the 118 children whom participants reported slept under ITNs, 36 were less than five years old.

Of the 119 participants who reported sleeping under programme-provided ITN the night prior to the survey, 115 (97%, CI: 94%–100%) reported that they slept under the pITN seven days a week. Nine participants reported sleeping under a non-programme-provided ITN every night.

Seventeen participants (12%, CI: 6%–18%) reported sleeping under any ITN fewer than seven nights a week or not at all. Among this group, the most frequent reported reason for not using an ITN was because it was too hot (8/17). Other reported reasons included: ITN was unavailable (6/17) or too difficult to mount (1/17), there were too few mosquitoes (2/17), participant slept away from home (2/17) or forgot (1/17). Of those who report sleeping under an ITN seven days a week (N = 124), the most frequent reasons for occasionally not sleeping under an ITN were that it was too hot (25/124) or that the participant slept away from home for work, visiting relatives, or for funerals (12/124).

### Perceptions of ITN effectiveness

113/142 (80%, CI: 73%–87%) of participants agreed that ITNs are used to prevent malaria, 81/142 (57%, CI: 49%–65%) said ITNs were useful for keeping out mosquitoes, and 26/142 (18%, CI: 12%–24%) thought ITNs were used to prevent fever.

### Home visits [please see Table 2]

**Table 2:** Results of random unannounced home visits for interview and ITN inspection

**Home visits to recipients of programme ITNs**	**N = 13**	**95% Conf. Int.**
# of household ITNs found matched self-report	13/13 (100%)	-

Total # of ITNs found in all visited households	32	-

Mean ITNs per household	32/13 = 2.5	-

ITNs in working order and mounted	28/32 (88%)	(72 – 95)
ITNs torn and not mounted	1/32 (3%)	-
ITNs torn and still mounted	3/32 (9%)	-

Households in which none of ITNs were mounted	3/13 (33%)	(8 – 50)

Approximately 10% (n = 13) of participants' households were visited. In all cases, the number of ITNs found in the home matched the number the participant had reported having received from the programme. However, in 3/13 (33%, CI: 8%–50%) households none of the ITNs was mounted. Most ITNs were in working order, however 4 of 32 total observed ITNs were found to be torn, and 3 of these torn ITNs were still mounted.

## Discussion

There is high self-reported retention and appropriate use of ITNs distributed by a community-based outpatient HIV clinic in Rakai, Uganda. In this rural region with moderate seasonal falciparum malaria, 75% of surveyed households had no ITNs except from the HIV treatment programme; in these households no other organization had provided ITNs, nor had any been purchased, although ITNs are available for sale in the community.

Study participants, who were the initial recipients of households ITNs, were the most likely person to report having slept under ITNs. While there is data suggesting that decreasing the burden of malaria in HIV-positive persons improves clinical outcomes [9], the majority of participants in this study were also receiving chronic trimethoprim-sulphamethoxazole prophylaxis, which has anti-malarial properties. Because of this, HIV patients on chronic trimethoprim-sulphamethoxazole prophylaxis, especially children, may be at less risk of malaria-related morbidity and mortality than some HIV-negative members of their households, who are not taking trimethoprim-sulfaphamethoxazole. [10]

Approximately half of pregnant women in participant households reportedly did not sleep under available ITNs. The benefits of ITN distribution to HIV patients could be enhanced by instructing recipients to have pregnant women and children under five also sleep under ITNs. Further investigation of co-sleeping patterns, provision of enough ITNs for all household members, and more detailed assessment regarding perceptions of who benefits most from ITN use may be beneficial. Additionally, families living within 300 meters of a household with an ITN have been shown to have reductions in child mortality, moderate anaemia, and high density parasitaemia. [11] ITN provision to HIV-affected individuals in high HIV-prevalence communities may serve an important role in a strategy of 'herd protection' if high ITN coverage is achieved in targeted communities.

This survey was limited to reports from those randomly selected individuals who agreed to participate. In addition, participants were interviewed by staff from the same programme which distributed ITNs and provided HIV care. Thus there was high potential for social desirability bias which may explain the difference between reported 123/128 (96%, CI: 93%–99%) proportion of programme ITNs in use by at least one household member and the 10/13 (77%, CI: 8%–50%) proportion of ITNs actually found to be mounted when inspected during home visits (although the total number of observations was small, and 95% confidence intervals nearly overlap). More home visits would have been advantageous, but were not possible given resource constraints. Regardless, ITN hang-up campaigns might remedy the fact that some ITNs were found un-mounted in homes. [12]

## Conclusion

There was good self-reported retention and continued appropriate use of ITNs distributed as a part of a PEPFAR-supported community-based outpatient HIV care programme in rural Uganda. Data collected through interviews corresponded moderately well with true ITN usage as demonstrated in unannounced home visits. More than 50% of pregnant women and many children below five years of age were sleeping under ITN in net-owning households, suggesting that education messages encouraging vulnerable people to sleep under ITNs can be effective when coupled with ITN provision. Provision of ITNs to HIV-positive individuals provides personal health benefits, and in this rural region with low ITN ownership, has increased overall ITN coverage not only for HIV-positive individuals but for their family members as well.

## Competing interests

The authors declare that they have no competing interests.

## Authors' contributions

LC and JK conceived of and designed the study with SJR. RMG supervised and IJ implemented the study. Field activities were coordinated by JL, including home visits. JS managed data and study records. LAM, LC, and JK performed the statistical analysis and drafted the manuscript. All were supervised by SJR. All authors read and approved the final manuscript.
